# Healthcare-associated bloodstream infections in critically ill patients: descriptive cross-sectional database study evaluating concordance with clinical site isolates

**DOI:** 10.1186/s13613-014-0034-8

**Published:** 2014-11-25

**Authors:** Nick Culshaw, Guy Glover, Craig Whiteley, Katie Rowland, Duncan Wyncoll, Andrew Jones, Manu Shankar-Hari

**Affiliations:** 1 Department of Intensive Care Medicine, Guy’s and St Thomas’ NHS Foundation Trust, 1st Floor, East Wing, St Thomas’ Hospital, London, SE1 7EH, UK; 2 Division of Asthma, Allergy and Lung Biology, King’s College London, London, SE1 9RT, UK

**Keywords:** Bacteremia, Intensive care units, Nosocomial infections

## Abstract

**Background:**

Healthcare-associated bloodstream infections are related to both increased antibiotic use and risk of adverse outcomes. An in-depth understanding of their epidemiology is essential to reduce occurrence and to improve outcomes by targeted prevention strategies. The objectives of the study were to determine the epidemiology, source and concordance of healthcare-associated bloodstream infections with clinical site isolates.

**Methods:**

We conducted a descriptive cross-sectional study in critically ill adults admitted to a tertiary semi-closed intensive care unit in England to determine the epidemiology, source and concordance of healthcare-associated bloodstream infections with clinical site isolates. All nosocomial positive blood cultures over a 4-year study period were identified. Pathogens detected and concordances with clinical site are reported as proportions.

**Results:**

Contaminant pathogens accounted for half of the isolates. The most common non-contaminant pathogens cultured were *Pseudomonas* spp. (8.0%), *Enterococcus* spp. (7.3%) and *Escherichia coli* (5.6%). Central venous catheter-linked bloodstream infections represent only 6.0% of the positive blood cultures. Excluding contaminants and central venous line infections, in only 39.5% of the bloodstream infections could a concordant clinical site source be identified, the respiratory and urinary tracts being the most common.

**Conclusions:**

Clinical practice should focus on a) improving blood culture techniques to reduce detection of contaminant pathogens and b) ensuring paired clinical site cultures are performed alongside all blood cultures to better understand the epidemiology and potential implications of primary and secondary discordant health-care associated bloodstream infections.

## Background

Healthcare-associated infections (HCAIs) [1] are common in patients managed in a critical care setting (ICU) [2]-[6]. HCAIs are often associated with indwelling devices and/or breach of natural barrier defence mechanisms and thus are potentially preventable with modifications in practice [7]-[12]. HCAI bloodstream infections in an ICU setting (ICU-BSIs) refer to the detection of pathogenic organisms in blood culture 48 h or more following ICU admission [1],[5]. Similar to other HCAIs in an ICU setting, ICU-BSIs are common and patients who develop ICU-BSIs are at higher risk of adverse outcomes [2]-[5],[13],[14].

ICU-BSI can either occur secondary to the dissemination of pathogens from a primary focus of infection at a clinical site into the bloodstream, or can be primary where the source of infection is unclear. The common clinical site foci for secondary ICU-BSI are the respiratory, gastrointestinal and urinary tracts [3]-[6],[15]-[17]. Although, several studies have examined the epidemiology and the prognostic utility of ICU-BSI, a useful addition to this literature would be the assessment of concordance between the pathogens isolated from ICU-BSI and those isolated from clinical site sampling that define HCAI. To address this question, we conducted a descriptive study of positive blood cultures identified in adult critically ill patients 48 h or more following admission to a single centre tertiary ICU in England over a 4-year period. The study objectives were to describe the epidemiology of pathogens identified in ICU-BSI and to determine the proportions of secondary ICU-BSI by identifying the most likely clinical source of ICU-BSI and the proportions of primary ICU-BSI where no corresponding clinical site could be identified.

## Methods

### Setting

Guy's and St. Thomas' NHS Foundation Trust (GSTT; London, England) is a 1,150-bed teaching hospital with a semi-closed, mixed medical and surgical ICU (approximately 70% medical and 30% surgical admissions). The infection prevention and control practices in our ICU areas include the Department of Health ventilator-associated pneumonia (VAP) prevention bundle [18], antibiotic guidelines with a course limited to 5 days [19], screening for MRSA and gentamicin-resistant Gram-negative bacilli on admission, universal chlorhexidine daily bathing (since 2007 and throughout the entire duration of the study) and limiting the duration of peripheral lines to 3 days. The ICU does not use selective digestive tract decontamination, but chlorhexidine oral gel is used as part of the VAP prevention bundle. Blood cultures are not taken from indwelling vascular access catheters. Precautions against catheter-linked bloodstream infections (CLBSIs) included the use of antimicrobial-impregnated central venous catheters, chlorhexidine-impregnated dressing sponges and a daily checklist that incorporated a reminder to review the need to remove or change the central catheter. Clinical care of the patients and the initial decision to send microbiological investigations was at the discretion of the treating physicians.

### Study population and blood culture database

Between December 2009 and November 2013, consecutive ICU admissions with an ICU length of stay greater than 48 h and who had at least one positive blood culture result greater than 48 h post-ICU admission were detailed prospectively in a quality improvement database (Access, Microsoft, New York, USA). Since inception, the database was updated weekly directly from the institution's central microbiological system. The hospital microbiology reports use a combination of colony morphology, Gram-staining and API® identification system (Biomérieux, Durham, USA) to identity and quantify (semi-quantitative) the microorganism identified in blood culture and clinical site samples. All positive blood cultures were then reviewed by one of four ICU physicians (DW, AJ, GG or MSH) with reference to electronic clinical records, imaging and other laboratory and microbiological data to determine the source of infection. Where there was uncertainty, a consensus view was obtained with review by two or more of the participating physicians. The data collection and analysis for this study were part of an ongoing GSTT hospital-approved clinical quality improvement and audit process, for which informed consent was waived for collection and reporting of anonymised data.

### Definitions

CLBSIs were identified from blood culture results and microbiological data as the database was updated. The CLBSI group includes both catheter-associated bloodstream infection (CABSI) and catheter-related bloodstream infection (CRBSI) defined using the accepted standard definitions [1],[11]. The non-CLBSI results were then categorised according to the species cultured. Results yielding contaminant species only were classified as ‘contaminants’ and results yielding potentially pathogenic species were classified as ICU-BSI. Microbiology culture results for clinical site samples taken within 48 h of the positive blood culture were then analysed in an attempt to identify primary sites of infection. When no clinical site culture results were positive, then the blood culture result was defined as a primary ICU-BSI. Blood culture results that did have positive clinical site culture results were classified as secondary ICU-BSI. Detection of pathogens with similar antibiogram in ICU-BSI and in clinical site samples taken within 48 h of the blood culture defined a concordant secondary ICU-BSI [1]. If a pathogen was present in blood culture and there was a clinical site isolate which did not satisfy the definition of concordance described above, then the results were defined as discordant secondary ICU-BSI. In line with accepted definitions, the following blood culture and clinical site isolates were classified as contaminants unless there were two or more concordant culture positive results and/or clinical site of infection identified by a treating physician: diphtheroids (*Corynebacterium* spp.), *Bacillus* (not *Bacillus anthracis*) spp., *Propionibacterium* spp. and coagulase-negative *Staphylococci* (CoNS).

### Statistics

The clinical details of the study cohort are presented as mean and standard deviation for continuous variables and as median and interquartile range for categorical variables. The microbiological results are presented as number and proportions with a denominator for calculating proportion presented in the tables.

## Results

### Study cohort

The case definition used identified 326 patients with one or more positive blood cultures. The mean (SD) age of the cohort was 60.8 (16.4) years and 65.2% were male. The mean (SD) APACHE II score on admission was 19.2 (6.3) and the median (interquartile range (IQR)) admission day total sequential organ failure assessment (t-SOFA) score was 6 [4],[9]. The ICU and hospital mortality of the study cohort was 31.5% and 38.3%, respectively. The median (IQR) ICU LOS was 14 [6],[20] (Table 1). The respiratory-, cardiovascular- and renal-specific SOFA scores, organ support provided and other characteristics of patients on the day of positive blood culture are shown in Table 2.

**Table 1:** Study population

**Parameter**	**Results**	**Missing values** ***n*** **(%)**
Age (years)	60.0 (15.4)	0
Sex (female:male)	110:216	0
Medical:surgical	286:40	1 (0.2)
Source of admission:		1 (0.2)
Ward *n*(%)	91 (27.2)	
Other hospital *n*(%)	94 (28.4)	
Emergency department *n*(%)	51 (15.4)	
Theatre	40 (12.1)	
Other *n*(%)	50 (15.3)	
APACHE II score (admission day)	18.8 (5.5)	2 (0.006)
Total SOFA score (admission day)	7 (4 to 10)	1 (0.2)
Hospital LOS prior to ICU admission (days)	1 (0 to 6)	3 (0.6)
Critical care LOS prior to first blood culture positive culture positive (days)	9 (5 to 16)	20 (6.1)
Critical Care LOS (days)	24 (13 to 40)	28 (5.4)
Ventilator days	23 (11 to 39)	14 (4.3)
ICU mortality/hospital mortality (%)	34.7/39.8	1 (0.2)

**Table 2:** Characteristics of patients on the day of positive blood culture

**Parameter**	**Results**	**Missing values** ***n*** **(%)**
Number of lines *in situ*		0
*n* =1	179 (54.9)	
*n* =2	121 (36.6)	
*n* =3	23 (7.0)	
*n* =4	3 (0.9)	
Respiratory SOFA score		0
0	8 (2.5)	
1	27 (8.4)	
2	116 (36.2)	
3	137 (42.5)	
4	38 (11.8)	
Mechanical ventilation	287 (88)	0
Airway		0
Endotracheal tube	169 (51.8)	
Tracheostomy	128 (39.2)	
Mask	29 (8.9)	
Renal SOFA score		
0	105 (33.0)	
1	41 (12.4)	
2	31 (9.4)	
3	45 (13.6)	
4	104 (31.4)	
Renal replacement therapy	136 (41.7)	0
CVS SOFA score		
0	35 (10.7)	
1	100 (30.2)	
2	0	
3	51 (15.4)	
4	140 (42.3)	

### Blood culture results

**Figure 1:**
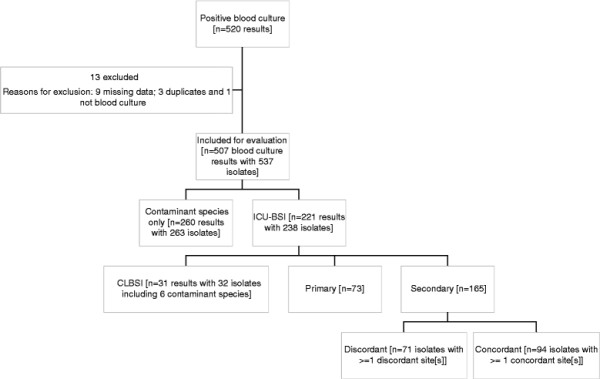
Shows the flow diagram for blood culture results.

Over the 48-month study period 507 positive blood culture results were recorded, with 537 individual isolates identified from these positive results. Of the blood cultures, 5.7% (*n* =29) contained more than one isolate with 28 identifying two isolates and one blood culture identifying three isolates (Table 2). Of the organisms detected, 94.4% (*n* =507) were bacterial. Gram-positive bacteria (GPB) were the predominant organism isolated (62.0% (*n* =333)) followed by Gram-negative bacteria (GNB) (32.4% (*n* =174)) and fungi (5.6% (*n* =30)). Contaminant species were the sole organisms cultured in 51.3% (260/507) of the positive blood cultures (Figure 1).

### CLBSI

CLBSI accounted for 6.0% (32/537) of all the positive blood culture isolates identified by our case definition and 12.2% (32/262) of all the ICU-BSIs when contaminant pathogens not meeting CLBSI definitions were excluded. During the 48-month study period, there were 5,982 patients with a central venous catheter (CVC) resulting in a total of 36,838 CVC days. The 3-month rolling average mean CRBSI rate was 0.66/1,000 CVC days and CLBSI rate was 1.19/1,000 CVC days. GPB and GNB accounted for most of the CLBSIs (Table 3). The majority of CLBSIs related to internal jugular lines (63%) compared to 19% femoral, 9% subclavian and others (peripherally inserted central catheters). The median (IQR) ICU length of stay before a CLBSI occurred was 8 (3 to 14) days, and the median duration of insertion for CLBSI lines were internal jugular 132 (120 to 168) hours, femoral 98 (96 to 115) hours and subclavian 670 (397 to 702) hours. Recognised risk factors for CLBSI were present in many patients (mechanical ventilation 74%, blood transfusion 54%, steroids 23% and parenteral nutrition 21%).

**Table 3:** Types and species of organisms isolated in all positive blood cultures, in CLBSI and in primary ICU-BSI

**Organism isolated**	**All isolates (*n*** **=537)**	**CLBSI (*n*** **=32**^**a**^**)**	**Primary ICU-BSI (*n*** **=73)**
Gram-positive	333 (62.0)	10 (1.9)	20 (27.4)
*Staphylococcus* species (CoNS)	246 (45.8)	6 (1.1)	-
Other contaminant species	26 (4.8)	-	-
Vancomycin-sensitive *Enterococcus*	31 (5.8)	2 (0.4)	12 (16.4)
Methicillin-sensitive *S. aureus*	13 (2.4)	1 (0.2)	2 (2.7)
Vancomycin-resistant *Enterococcus*	8 (1.5)	1 (0.2)	4 (5.5)
*Streptococcus* other species	4 (0.7)	-	2 (2.7)
Methicillin-resistant *S. aureus*	1 (0.2)	-	-
*Streptococcus pneumoniae*	1 (0.2)	-	-
Unidentified probable contaminant	3 (0.6)	-	-
Gram-negative	174 (32.4)	13 (2.4)	45 (61.6)
*Escherichia coli*	32 (5.6)	1 (0.2)	7 (9.6)
*Pseudomonas aeruginosa*	43 (8.0)	6 (1.1)	6 (8.2)
Other	35(6.5)^b^	1 (0.2)^c^	13 (17.8)
*Klebsiella* species	27 (5.0)	4 (0.7)	10 (13.7)
*Serratia* species	18 (3.4)	-	5 (6.8)
*Enterobacter* species	19 (3.5)	1 (0.2)	4 (5.5)
Fungi	30 (5.6)	9 (1.7)	8 (11.0)
*Candida* species	30 (5.6)	9 (1.7)	8 (11.0)

^a^Denominator for calculating proportions was the total number of positive blood culture isolates (*n* =537). ^b^Includes *Proteus*, *Bacteroides*, *Citrobacter*, *Morganella* and *Stenotrophomonas* species. ^c^*Proteus mirabilis*.

### Primary and secondary ICU-BSI

A total of 238 isolates, identified from 217 non-CLBSI, non-contaminant blood cultures (with *n* =19 with two and *n* =1 with three isolates) were compared with clinical site cultures to identify primary and secondary ICU-BSI (Table 3). Secondary ICU-BSIs were more common than primary ICU-BSIs (*n* =165 (69.3%) and *n* =73 (30.7%), respectively). The most common pathogens isolated in primary ICU-BSI are detailed in Table 3.

Amongst the secondary ICU-BSIs, concordant secondary ICU-BSIs were more common than discordant secondary ICU-BSIs (94/165 (57.0%) and 71/165 (43.0%), respectively). The most common clinical site of concordant secondary ICU-BSI was the respiratory tract, and the pathogens isolated most often were *Pseudomonas* spp., *Escherichia* coli and *Staphylococcus aureus*. Amongst the discordant ICU-BSI, the most common clinical site was again the respiratory tract. The most common pathogens isolated in ICU-BSI with discordant clinical site isolate were *Klebsiella* spp., *Enterococcus faecium* and *E. coli* (Table 4).

**Table 4:** Attributed sources of secondary ICU acquired bloodstream infections

**Source of blood culture isolate**	**Concordant isolate** ***n*** **(%)**	**Discordant isolates** ***n*** **(%)**
Pulmonary (BAL or sputum)	50(53.2)	50(70.4)
Renal/urinary tract	22(23.4)	25(35.2)
Skin and soft tissue	8(8.5)	11(15.5)
Other (aortic valve graft, joint aspirate, limb tissue)	8(8.5)	10(14.1)
Intra-abdominal (fluid or surgical swab)	10(10.6)	6(8.5)
Surgical wound site	3(3.2)	5(7.0)

Note: Isolates have been attributed to multiple sources where concordant cultures exist for more than one organ system within the 48-h window before and after the blood culture was taken. Denominator for calculating proportions were the total number of concordant isolates (*n* =94) and discordant isolates (*n* =71). BAL, bronchoalveolar lavage.

## Discussion

In an adult ICU setting, CLBSIs accounted for 6.0% of all positive blood cultures and 12.2% of all ICU-BSIs with a CRBSI rate of 0.66/1,000 catheter days, much lower than the published literature. Fungaemia accounted for a significant minority of positive blood cultures (5.6% of all positive cultures and 11.5% of all ICU-BSIs). Amongst ICU-BSIs, a discordant clinical site or no clinical site could be identified in the majority (60.5%) of the positive blood cultures. Secondary concordant ICU-BSIs were more common than secondary discordant ICU-BSIs. The respiratory tract was the most common site of infection and GNB were the predominant isolates in the ICU-BSIs identified.

Inability to identify a clinical site source (primary ICU-BSI) and discordant secondary ICU-BSI in 60% of the study population are key findings addressing the study objective and could have several possible explanations. Firstly, these isolates may not represent true ICU-BSIs but detection of potentially pathogenic organisms colonising the skin site from where blood cultures were taken, i.e. ‘contamination with potentially pathogenic organisms’. Secondly, paired sampling of suspected clinical site and blood culture was not mandated in our study, thus falsely identifying primary ICU-BSI by not detecting a corresponding clinical site infection. Thirdly, there were occult sources of infection that were not identified or suspected and therefore not cultured, potentially falsely identifying either primary ICU-BSI or non-concordant secondary ICU-BSI (if original blood culture had multiple isolates). Finally, both primary ICU-BSI and secondary non-concordant ICU-BSI (multiple isolates) may reflect infection from a source that is not feasible to sample - the most obvious being translocation from the gastro intestinal (GI) tract as nearly 83% of all clinical isolates could be detected first in microbiological sampling of the GI tract [12].

The key design strength is the consistent temporal review process with a limited number of experienced reviewers working to agreed criteria with access to contemporaneous medical records. The study reports all positive blood cultures isolated in a large critical care service with high compliance rates in HCAI prevention strategies and antimicrobial use [19] thus providing a representative sample from which inferences about practice interventions could be made. Our results have similarities with the report from another tertiary-level critical care unit in London [21], giving it further validity. The study setting is a large mixed medical and surgical ICU with high compliance with infection prevention and control measures.

Our study has a number of limitations. It is a retrospective, descriptive database review of bacteraemia cases. Although the blood culture practices were standardised, neither the sampling method nor the paired clinical site samples were mandated, thus potentially introducing bias from clinical site samples processed due to ease. As a descriptive study, we have not adjusted for potential confounders. However, the aim was to report an updated epidemiology of blood cultures from an ICU in England and to explore the concept of concordance between ICU-BSI and clinical site of nosocomial infections.

Along with detection of CoNS spp. as the commonest contaminant, the most common pathogenic GPB isolates in our study were *S. aureus* spp. and *Enterococcus* spp., which correspond to the published literature [4],[16],[21]-[26]. The most common pathogenic GNB isolates in our study were *E. coli*, *Klebsiella* spp. and *Pseudomonas aeruginosa* spp. which are similar to other ICU studies [4],[16],[21]-[26]. Primary ICU-BSI was observed in 31.6% of our cohort, which is similar to the study by Stohl et al. [25] but higher than some reports [22],[27] and may be partially explained by our study design, with paired cultures not mandated. Nearly 11% of ICU-BSI isolates were fungi and most often *Candida* species, which is similar to a large multicentre study of nosocomial BSI from the United States [14]. In primary ICU-BSI, 70% of isolates were enteric pathogens implying either an occult GI source of infection or GI translocation, which corresponds to published literature on the role of GI tract in pathogenesis of nosocomial infections [12],[28]. Corona et al. in a single centre ICU surveillance of blood cultures report that nearly 90% of bacteraemia episodes satisfy the HCAI definitions [21]. The proportion of fungaemia in the study was 3%, in 45% of patients with HCAI the source of infection was GI or respiratory, and there was an excess of contaminant cultures, which are comparable results to our study [22]. Finally, CLBSI rate in our study was 1.19/1,000 CVC days and the CRBSI rate was 0.66/1,000 CVC days, both significantly lower than the mean rates reported for adult ICUs in England [11]. This is reflective of the multifaceted interventions in place in our ICU, which aims to optimise sterile insertion technique and ongoing line care, whilst avoiding femoral access and the prolonged dwell time of unnecessary lines.

More than 50% of positive blood cultures isolated were contaminant species, which is often related to poor technique and results in inappropriate empiric antibiotic therapy with its attendant challenges. Consequently, efforts to reduce ‘contaminant rates’ in critically ill patients would seem a worthwhile strategy. Contaminant rates in blood culture are usually reported as between 0.6% and 10% when the denominator used to calculate the rates is all blood cultures sent to the lab. In our study, the high rate of contaminants is artificially high as our denominator is all positive blood cultures and not all blood cultures sent to the lab. When applying the same definition as published literature the contaminant rates seen in our ICU was 2.3% (between July and September 2014, unpublished data from local audit).

A number of strategies have been proposed to reduce the contaminant rates, as well as to reduce true bacteraemias. A key intervention to reduce blood culture contamination in ICU patients is the use of chlorhexidine baths, which has been implemented in our ICU before the study period as part of routine clinical care [29]-[31]. Another potential intervention is selective oral and/or digestive tract decontamination (SDD/SOD). Aside from chlorhexidine for oral application as part of the routine VAP bundle, SDD/SOD is not used in our ICU. The challenges with SDD/SOD include (but are not limited to) costs, potential for generating resistance pathogens, inconsistent effect on mortality and potential for altering the pathogen ecology of the ICU where the intervention is implemented, and questions remain regarding the ideal combination of drugs for this intervention [20],[32].

The primary ICU-BSI and discordant secondary ICU-BSI represent either inadvertent sampling of clinically irrelevant organisms (akin to contaminants) or yet-to-be-determined secondary sources of infection; both have implications regarding antibiotic course duration and risk of recurrence. Therefore, in the presence of primary or particularly discordant secondary ICU-BSI, the clinician should actively consider investigations to exclude occult infections or repeat the microbiology surveillance for the most likely clinical site. Thus further research is required to explore the implications of isolating either a secondary discordant ICU-BSI or a primary ICU-BSI where paired cultures of potential sites have been sent, to answer the potential additional morbidity and antibiotic use in this population. This question is relevant as similar to culture positive and culture negative sepsis [3], the outcomes might differ between concordant and discordant blood cultures depending on treatment variables.

## Conclusions

Contaminant species are frequently isolated in blood cultures performed in ICU patients, but rarely represent true ICU-BSI thus should be a focus for future quality improvement strategies. CLBSI and fungaemia represent only a small proportion of ICU-BSI at our institution. In concordant ICU-BSIs, respiratory and urinary tract infection were the most common sources. In trying to reduce the burden of ICU-BSI, the focus should be on implementing strategies to prevent respiratory and urinary tract infections in ICU, alongside contaminant reduction.

## Competing interests

The authors declare that they have no competing interests.

## Authors’ contributions

MSH conceived and designed the study. DW, AJ, GG and MSH contributed to the database of positive blood culture results used in the study. CW maintained the database. CW, NC and KR extracted data for analysis. All authors had full access to all of the data (including statistical reports and tables) in the study and take responsibility for the integrity of the data and the accuracy of the data analysis. All authors have read and approved the final manuscript. NC, AJ and CW are guarantors of the data.
